# Combined Motivational Interviewing and Ecological Momentary Intervention to Reduce Hazardous Alcohol Use Among Sexual Minority Cisgender Men and Transgender Individuals: Protocol for a Randomized Controlled Trial

**DOI:** 10.2196/55166

**Published:** 2024-04-05

**Authors:** Carolyn Lauckner, Bryce Puesta Takenaka, Fidelis Sesenu, Jaime S Brown, Sally J Kirklewski, Erin Nicholson, Kimberly Haney, Reuben Adatorwovor, Donte T Boyd, Keisa Fallin-Bennett, Arjee Javellana Restar, Trace Kershaw

**Affiliations:** 1 Center for Health Equity Transformation University of Kentucky Lexington, KY United States; 2 Department of Social and Behavioral Sciences Yale School of Public Health New Haven, CT United States; 3 Department of Biostatistics University of Kentucky Lexington, KY United States; 4 College of Social Work The Ohio State University Columbus, OH United States; 5 Center for Interdisciplinary Research on AIDS Yale University New Haven, CT United States; 6 Department of Family and Community Medicine University of Kentucky Lexington, KY United States; 7 Department of Epidemiology Department of Health Systems and Population Health University of Washington Seattle, WA United States

**Keywords:** alcohol use, sexual minority, transgender, young adults, mobile health, mHealth, HIV risk behaviors, sexual risk behaviors, motivational interviewing, ecological momentary interventions, mobile phone

## Abstract

**Background:**

Sexual minority cisgender men and transgender (SMMT) individuals, particularly emerging adults (aged 18-34 years), often report hazardous drinking. Given that alcohol use increases the likelihood of HIV risk behaviors, and HIV disproportionately affects SMMT individuals, there is a need to test interventions that reduce hazardous alcohol use and subsequent HIV risk behaviors among this population. Ecological momentary interventions (EMIs), which use mobile phones to deliver risk reduction messages based on current location and behaviors, can help to address triggers that lead to drinking in real time.

**Objective:**

This study will test an EMI that uses motivational interviewing (MI), smartphone surveys, mobile breathalyzers, and location tracking to provide real-time messaging that addresses triggers for drinking when SMMT individuals visit locations associated with hazardous alcohol use. In addition, the intervention will deliver harm reduction messaging if individuals report engaging in alcohol use.

**Methods:**

We will conduct a 3-arm randomized controlled trial (N=405 HIV-negative SMMT individuals; n=135, 33% per arm) comparing the following conditions: (1) Tracking and Reducing Alcohol Consumption (a smartphone-delivered 4-session MI intervention), (2) Tracking and Reducing Alcohol Consumption and Environmental Risk (an EMI combining MI with real-time messaging based on geographic locations that are triggers to drinking), and (3) a smartphone-based alcohol monitoring–only control group. Breathalyzer results and daily self-reports will be used to assess the primary and secondary outcomes of drinking days, drinks per drinking day, binge drinking episodes, and HIV risk behaviors. Additional assessments at baseline, 3 months, 6 months, and 9 months will evaluate exploratory long-term outcomes.

**Results:**

The study is part of a 5-year research project funded in August 2022 by the National Institute on Alcohol Abuse and Alcoholism. The first 1.5 years of the study will be dedicated to planning and development activities, including formative research, app design and testing, and message design and testing. The subsequent 3.5 years will see the study complete participant recruitment, data collection, analyses, report writing, and dissemination. We expect to complete all study data collection in or before January 2027.

**Conclusions:**

This study will provide novel evidence about the relative efficacy of using a smartphone-delivered MI intervention and real-time messaging to address triggers for hazardous alcohol use and sexual risk behaviors. The EMI approach, which incorporates location-based preventive messaging and behavior surveys, may help to better understand the complexity of daily stressors among SMMT individuals and their impact on hazardous alcohol use and HIV risk behaviors. The tailoring of this intervention toward SMMT individuals helps to address their underrepresentation in existing alcohol use research and will be promising for informing where structural alcohol use prevention and treatment interventions are needed to support SMMT individuals.

**Trial Registration:**

ClinicalTrials.gov NCT05576350; https://www.clinicaltrials.gov/study/NCT05576350

**International Registered Report Identifier (IRRID):**

PRR1-10.2196/55166

## Introduction

### Background

Those who identify as sexual and gender minority individuals (eg, gay, bisexual, transgender, or queer individuals) are more likely to report hazardous drinking, defined as “drinking behavior (such as per episode, daily, or weekly) that reflects meaningful increases in risk of negative alcohol-related outcomes” [1] and to have alcohol use disorders (AUDs) than their cisgender and heterosexual peers [2-5]. Similarly, emerging adult (aged 18-34 years) sexual and gender minority individuals are at increased odds of being diagnosed with AUDs compared to older adults, with up to 44% meeting AUD criteria [3,6]. An extensive body of research suggests that the increased use of alcohol among young sexual and gender minority populations is attributable to interlocking structural underpinnings of stigma, homophobia, transphobia, and other manifestations of violence against queer people [5,7-9]. These trends are concerning because multiple studies have found that alcohol use increases the likelihood of acquiring HIV through various behaviors [10-13] and that HIV disproportionately affects emerging adult sexual minority cisgender men and transgender (SMMT) individuals [14]. Consequently, individuals who have reported unhealthy levels of alcohol use also were likely to experience interference with their decision-making with regard to adhering to pre-exposure prophylaxis (PrEP) [15], which can prevent HIV. Thus, it is essential to test interventions for reducing alcohol use among SMMT individuals as a means of preventing HIV among these populations considered to be at high risk.

A recent systematic review found that motivational interviewing (MI) is a frequently used method for reducing alcohol use [16]. MI is a goal-oriented and patient-centered approach to motivating behavior change and increasing self-efficacy. While it has been effective for reducing alcohol use among gay and bisexual men [17-19], this review found only 1 alcohol-related MI intervention for transgender people [20], suggesting the need for more research with this population. Prior studies also suggest the effectiveness of MI being delivered through technology to support the reduction of alcohol use behaviors [21]. The Tracking and Reducing Alcohol Consumption (TRAC) intervention, recently tested among people with HIV and currently being tested (since July 2023) among young adult survivors of cancer, uses smartphones to deliver 4 to 8 weekly sessions of MI for reducing risky drinking. The sessions focus on identifying triggers for drinking and strategies for addressing these triggers. TRAC also incorporates daily smartphone-based alcohol monitoring (SAM) using mobile breathalyzers and surveys. While preliminary results are promising in terms of TRAC’s effectiveness and acceptability [22], there is potential for enhancing it by reinforcing content in real-time situations where people are more likely to experience triggers to use alcohol.

Ecological momentary interventions (EMIs), which use smartphones to deliver messages to reduce alcohol use and related risk behaviors during or before drinking events, can help to address triggers in real time. GPS tracking can determine when individuals visit places where they have previously reported drinking or triggers to drink, and then EMI messages can be delivered upon arrival to *prevent* hazardous alcohol use. However, this GPS-based approach has rarely been studied to date and has generally targeted individuals with AUDs (as opposed to those who engage in hazardous drinking but do not have an AUD) [23,24]. We have developed a mobile app for a previous observational study that uses GPS tracking to determine when emerging adult SMMT individuals visit *risky* *locations* and then delivers a survey asking about aspects of the location’s social and geographic context that may relate to drinking and other risk behavior.

### Objectives

The goal of the proposed study is to use this app to enhance TRAC by delivering messages that encourage participants to use strategies discussed during TRAC sessions when arriving at risky locations. When they leave these locations, they will complete a survey and a breathalyzer reading to collect event-level self-report and biological data on alcohol use and HIV risk. If their breathalyzer result indicates alcohol use, they will receive harm reduction messaging. It is expected that combining TRAC with EMI (Tracking and Reducing Alcohol Consumption and Environmental Risk [TRAC-ER]) will increase effectiveness by reinforcing topics discussed during sessions, providing in-the-moment messaging to address triggers, and collecting real-time alcohol use data.

We will conduct a randomized controlled trial (RCT) of the TRAC and TRAC-ER interventions, examining their effects on alcohol use and HIV risk behaviors among SMMT individuals compared with a SAM-only control group. We will examine 2 aims.

- Aim 1: assess the effects of intervention conditions on drinking days (primary end point), drinks per drinking day, and binge drinking episodes (secondary end points).
  Hypothesis:
  TRAC and TRAC-ER will lead to significantly fewer drinking days compared with a SAM-only control group, with TRAC-ER having the strongest effects.
- Aim 2: assess the effects of intervention conditions on HIV risk behaviors (unprotected sex, number of partners, concurrent sex and substance use, and PrEP nonadherence).
  Hypothesis:
  TRAC and TRAC-ER will lead to significantly fewer HIV risk behaviors compared with a SAM-only control group, with TRAC-ER having the strongest effects.

## Methods

### Study Design

We will conduct a 3-arm RCT of TRAC and TRAC-ER (n=405 HIV-negative SMMT individuals; n=135, 33% per arm), with the following conditions: (1) SAM-only comparison group, (2) SAM+TRAC (no EMI messaging), and (3) SAM+TRAC-ER (participants receive EMI messaging when visiting risky locations).

Breathalyzer results and daily self-reports will be used to assess the primary and secondary outcomes of drinking days, drinks per drinking day, binge drinking episodes, and HIV risk behaviors. Additional assessments at baseline (T1), 3 months (T2), 6 months (T3), and 9 months (T4) will evaluate exploratory long-term outcomes, including diagnoses of HIV or other sexually transmitted infections (STIs).

### Participants and Recruitment

#### Overview

Participants will be recruited using web-based and community-based recruitment, as well as through health clinics that serve SMMT individuals. A combination of venue-based and purposive sampling approaches appropriate for accessing populations considered to be hard to reach and at risk will be used to recruit participants [25-27]. Previous intervention studies with young adult SMMT populations have found 6-month dropout rates of 15% to 25% [28-30]. To reach our target final sample (n=324; refer to the Sample Size Justification subsection), we will recruit 405 individuals, accounting for an estimated 20% dropout rate based on prior literature.

#### Health Clinics

All patients presenting for appointments at participating clinics will be screened using a preprogrammed tablet computer to determine eligibility. The principal investigators will provide instruction to clinic staff regarding screening patients and distribute quick-reference study information sheets to keep in offices. The study coordinator will be available during business hours to help clinic staff as needed.

#### Web-Based and Community-Based Recruitment

Our previous studies have successfully used a variety of recruitment methods to reach SMMT individuals, which we will also leverage in the proposed study: flyer outreach (in locations frequented by SMMT individuals), social media recruitment (including paid and free posts on Instagram, Facebook, Reddit, and dating apps), a study website, and institutional collaborations. All advertisements will direct participants to the web-based screener. We will use a community advisory board, comprising members representing different subsets of the SMMT population, to obtain further guidance regarding ideal participant recruitment methods.

#### Inclusion and Exclusion Criteria

Participants will be eligible to participate if they meet the following inclusion criteria: (1) self-identify as a gender minority individual (eg, transgender man, transgender woman, nonbinary individual, agender individual, or gender fluid individual) or a sexual minority cisgender man (ie, assigned male sex at birth and identifies as a man with a sexual orientation other than heterosexual), (2) is aged between 18 and 35 years at the start of the study, (3) owns a smartphone, (4) is HIV-negative (confirmed through a test at baseline), (5) meets Centers for Disease Control and Prevention PrEP eligibility criteria (individuals will be eligible if they have had anal or vaginal sex in the past 6 months and have at least one of the following characteristics: a sexual partner with HIV, inconsistent condom and PrEP use, or a sexually transmitted disease diagnosis in the past 6 months; or if they inject drugs and have an injection partner with HIV or share needles, syringes, or other equipment to inject drugs) [31], and (6) screens positively for hazardous alcohol use. As most alcohol screening instruments rely on biological sex, we will use a combination of the Alcohol Use Disorders Identification Test–Concise (AUDIT-C), a validated 3-item screener for hazardous alcohol use [32], and a 1-item yes or no screening question to determine whether they engage in hazardous alcohol use (“Have you had 5 or more drinks on one occasion in the past year?”). This question was found to be a valid measure of hazardous alcohol use among gender-diverse individuals; despite it being a single item, this screening method performed better than the AUDIT-C for lower drink thresholds (eg, ≥4 drinks) in predicting hazardous alcohol use [33]. However, because this is still a new approach to assessing hazardous drinking and because we will also be enrolling cisgender individuals, we will consider AUDIT-C scores too; participants must also score ≥4 on the AUDIT-C to qualify [32].

Exclusion criteria include (1) not speaking English; (2) active psychosis or severe mental illness; (3) actively detoxifying from substances and needing medical supervision; or (4) a score of ≥20 on the Alcohol Use Disorders Identification Test [32], which indicates that participants exhibit increased or higher risk drinking behaviors.

Regardless of the recruitment method used, all participants will complete a web-based screener. If participants are determined to be eligible according to the screener, they will be informed of their eligibility and invited to provide their contact information if they wish to learn more about the study.

#### Sample Size Justification

Sample size determination was based on the primary outcome variable: number of drinking days in the past month across the repeated visits corresponding to T1, T2, T3, and T4 for the 3 groups (SAM only, SAM+TRAC, and SAM+TRAC-ER). Data on effect sizes from previous alcohol use interventions [34,35] and our preliminary data (within-participant correlation of 0.69) were used to derive the mean number of days of alcohol use and their SDs. With a final sample size of n=324 (after accounting for a 20% dropout rate with a total n=405), 108 (33%) participants in each group will give us at least 90% power at 5% type I error rate to detect a significant difference among the groups for an effect size of 0.071 when comparing the groups over the study period. This effect size is smaller than a *small* effect as defined by Cohen et al [36].

We expect our sample to have the following characteristics: 48.1% (195/405) cisgender men, 24.9% (101/405) transgender men, 20% (81/405) transgender women, and 6.9% (28/405) nonbinary individuals. Our sample will likely have a higher proportion of transgender participants compared to the general population due to recruitment within health clinics that provide care for transgender patients; we view this as a strength of our study, given the underrepresentation of gender minority individuals in previous alcohol intervention and HIV prevention research.

#### Enrollment

If participants are determined to be eligible according to their responses to the web-based screener, they will be informed of their eligibility and invited to provide their contact information if they wish to learn more about the study. If participants are ineligible, they will be informed of this upon completion of the screener. A member of the study team will contact eligible participants via their preferred contact method to complete an interview confirming eligibility. This interview is conducted via Zoom (Zoom Video Communications, Inc) and is included as protection against spam or false screener responses.

### Study Interventions and Procedures

#### Description of TRAC-ER Study App

The app developed for this study has been designed to track participants’ physical location through continuous GPS monitoring and to deliver assessments and messages at specific times of the day and in response to participants’ physical location. At baseline, all participants will be asked to list the locations where they most frequently engage, or are triggered to engage, in alcohol use, unprotected sex, or other HIV risk behaviors as part of an activity space assessment. For each listed location, they will provide information regarding the types of alcohol triggers they experience (eg, social, situational, or emotional). These locations will then be programmed into the app and given a geofence (a distance around the location such that when participants break the geofence, intervention-specific actions such as surveys or messages are delivered). Before each follow-up monitoring period, participants will complete a short questionnaire that lists the locations they identified in the activity space assessment, as well as locations where they reported drinking via the random breathalyzer reading requests during prior monitoring periods. They will have the opportunity to revise the list of locations, which will ensure that the triggered surveys are being delivered for locations most relevant to the participants.

**Figure 1:**
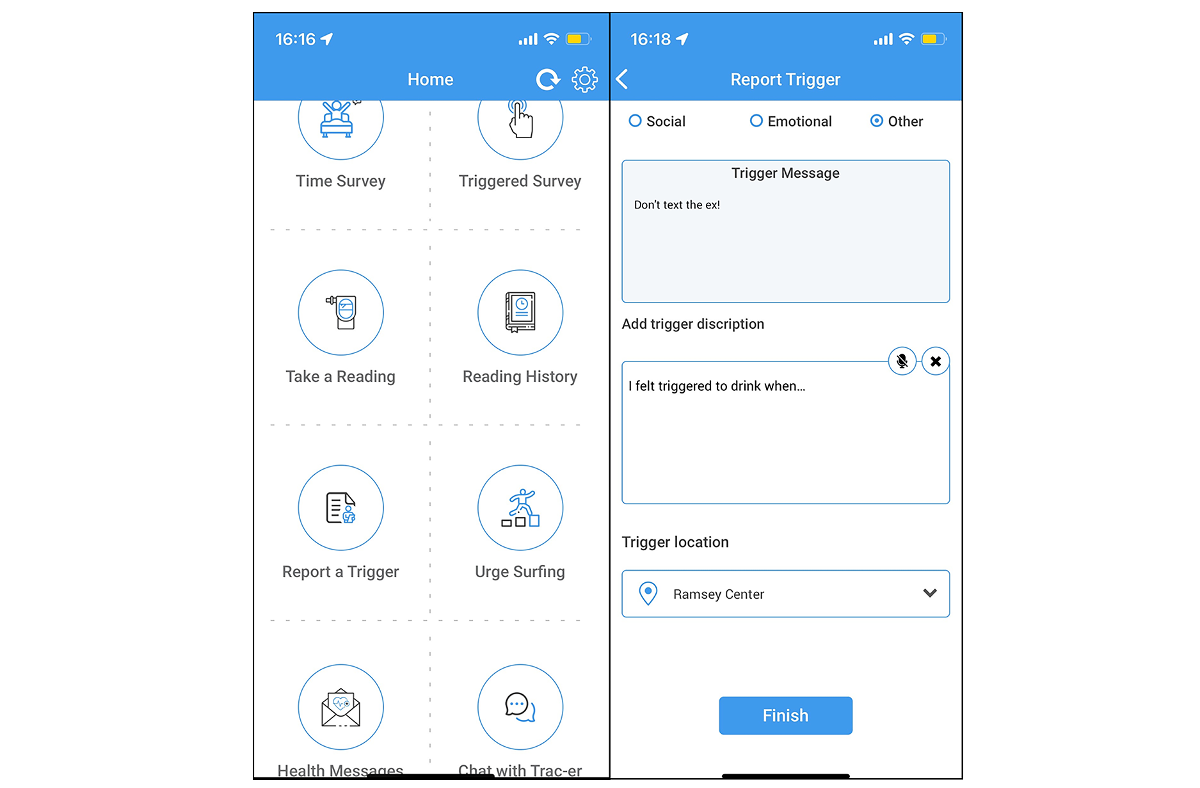
Tracking and Reducing Alcohol Consumption and Environmental Risk (TRAC-ER) app screenshots (app developed by Vista IT Solutions, LLC).

Every morning during the SAM periods, participants will receive a push notification asking them to report the number of drinks consumed in the previous day (if applicable), time spent drinking, alcohol withdrawal symptoms, and sexual risk behaviors. The app will also send requests for breathalyzer readings at least 2 times per day in response to visits to risky locations and through random requests. At the time of providing the breathalyzer reading, participants will also complete a brief mobile survey using the study app that includes questions related to their alcohol use. Participants can view a log of their breathalyzer readings in the app. Participants will have the option to report a trigger in the app and provide details about the trigger, including the location where the trigger was generated. Participants who are randomized to the TRAC-ER intervention will receive a harm reduction message based on the type of trigger (social, emotional, or other) they report. A guided breathing exercise and urge surfing exercise are included in the app for those in the TRAC and TRAC-ER conditions. Figure 1 presents a screenshot of the app. If, at any point during the monitoring periods, the app stops collecting GPS data, the study team will reach out to determine whether the participant accidentally turned off location tracking, lost their mobile phone, or is experiencing other technical difficulties.

#### Description of Study Arms

##### Overview

**Figure 2:**
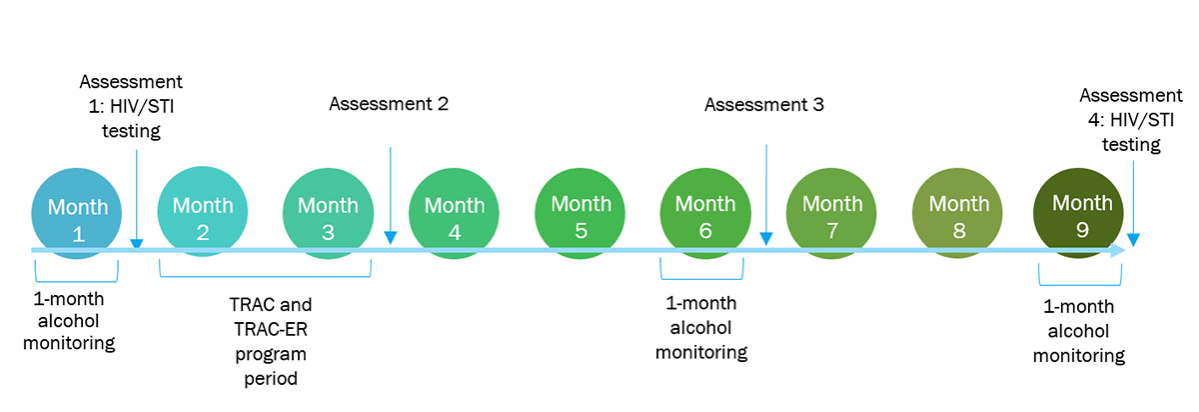
Participation timeline. STI: sexually transmitted infection; TRAC: Tracking and Reducing Alcohol Consumption; TRAC-ER: Tracking and Reducing Alcohol Consumption and Environmental Risk.

Participants will be enrolled in the study for 9 months, including the 2-month intervention period and multiple follow-up monitoring periods to assess long-term effects. All participants will begin with a 30-day monitoring period, which will establish baseline alcohol use through daily breathalyzer reading requests and surveys. After this 30-day period, they will complete additional baseline measures (T1) and then begin their assigned intervention, if applicable. After the interventions are delivered, participants will complete an additional self-report (T2). Subsequently, there will be 2 additional monitoring periods and assessments spread across the 9-month study period to assess long-term intervention effects, with some months involving no study activities. A depiction of the participation timeline is included in Figure 2, and details of study activities completed across the study timeline and RCT arms are provided in Table 1.

**Table 1:** Summary of research activities across the randomized controlled trial conditions.

Days and research activities		Control	TRAC^a^	TRAC-ER^b^
**1-30**				
	Morning survey: sent between 7 AM and 10 AM (based on participant preference)	✓	✓	✓
	Random breathalyzer reading and breathalyzer request survey: request at a random time between 11 AM and 5 PM and at a random time between 5 PM and 11 PM	✓	✓	✓
	Location survey and breathalyzer reading: request breathalyzer survey and breathalyzer reading when participant leaves a risky location	✓	✓	✓
**31-90**				
	Morning survey: sent between 7 AM and 10 AM (based on participant preference)	✓	✓	✓
	Random breathalyzer reading and breathalyzer request survey: request at a random time between 11 AM and 5 PM and at a random time between 5 PM and 11 PM	✓	✓	✓
	Location survey and breathalyzer reading: request breathalyzer request survey and breathalyzer reading when participant leaves a risky location	✓	✓	✓
**TRAC intervention**				
	Participant completes 4 TRAC sessions via video or telephone call on a weekly basis		✓	✓
**TRAC-ER in-app messages**				
	Deliver prevention message upon arrival at risky location			✓
	Deliver harm reduction message if, upon leaving a risky location, participants have a positive			✓
	Deliver a self-help message if participant taps *report a trigger* in the app			✓
**91-150**				
	No research activities			
**151-180**				
	Morning survey: sent between 7 AM and 10 AM (based on participant preference)	✓	✓	✓
	Random breathalyzer reading and breathalyzer request survey: request at a random time between 11 AM and 5 PM and at a random time between 5 PM and 11 PM	✓	✓	✓
	Location survey and breathalyzer reading: request breathalyzer request survey and breathalyzer reading when participant leaves a risky location	✓	✓	✓
**181-240**				
	No research activities			
**241-270**				
	Morning survey: sent between 7 AM and 10 AM (based on participant preference)	✓	✓	✓
	Two random breathalyzer readings and breathalyzer request survey: request at a random time between 11 AM and 5 PM and at a random time between 5 PM and 11 PM	✓	✓	✓
	Location survey and breathalyzer reading: request breathalyzer request survey and breathalyzer reading when participant leaves a risky location	✓	✓	✓

^a^TRAC: Tracking and Reducing Alcohol Consumption.

^b^TRAC-ER: Tracking and Reducing Alcohol Consumption and Environmental Risk.

##### SAM-Only Participants

Participants will complete alcohol self-monitoring tasks using the study app (daily surveys and breathalyzer readings) during months 1 to 3, month 6, and month 9. During these designated months, participants will receive prompts to complete morning surveys, random breathalyzer requests, and location surveys, as described in Table 1.

SAM-only participants will also complete the T1 to T4 assessments on the schedule illustrated in Figure 1.

##### TRAC Participants

TRAC participants will complete alcohol self-monitoring tasks and assessments following the schedule described for SAM-only participants. In addition, participants will receive the 4-session TRAC intervention. Individuals will meet for 30 minutes with an interventionist on a weekly basis via telephone or video chat, although the 6-week intervention period allows for some cancellations or rescheduling. Textbox 1 presents an overview of the 4-session TRAC intervention. Participants will be mailed a paper workbook to complete during the sessions. The SAM app will provide supplementary materials, including a triggers diary, a guided breathing exercise, and a record of the participants’ goals for reducing their drinking. After participants complete all intervention sessions, they will continue daily monitoring for the remainder of the 2-month intervention period. Participants will complete the T1 to T4 assessments following the schedule described for SAM-only participants.

**Session overview of the Tracking and Reducing Alcohol Consumption (TRAC) intervention.**

**Session 1**

- Introduction (5-8 minutes)
- Alcohol and health factsheet (10 minutes)
- Review current alcohol use (15 minutes)
- Complete change plan (10 minutes)
- Diaphragmatic breathing (8 minutes)
- Wrap up and schedule next session (5 minutes)

**Session 2**

- Recap of past session, check-in, and agenda setting (5 minutes)
- Review weekly drinking data and change goals from last week (10 minutes)
- Review strategies for managing triggers (20 minutes)
- Urge surfing skill (10 minutes)
- Wrap up and schedule next session (5 minutes)

**Session 3**

- Recap of past session, check-in, and agenda setting (5 minutes)
- Review weekly drinking data and change goals from last week (10 minutes)
- Strategies for reducing alcohol-related harm (20 minutes)
  Before known drinking occasions
  While drinking alcohol
  After drinking alcohol
- Wrap up and schedule next session (5 minutes)

**Session 4**

- Recap of past session, check-in, and agenda setting (5 minutes)
- Review weekly drinking data and change goals from last week (5-8 minutes)
- Review overall progress (5-8 minutes)
- Set goals and plan for going forward (10-15 minutes)
- Wrap up and give reminder of remaining study tasks (5 minutes)

##### TRAC-ER Participants

###### Overview

Participants will complete all aspects of the TRAC intervention condition. In addition to receiving triggered surveys when they leave a risky location, participants will also receive prevention messaging when they first arrive at a risky location. This messaging will be tailored based on the data collected regarding participants’ risky locations and the strategies to address triggers discussed during the MI intervention sessions. In addition, if participants return a breathalyzer reading >0.00%, they will receive harm reduction messaging encouraging them to engage in protective behaviors. These messages will continue throughout the 2-month intervention period, including after the 4 TRAC sessions have been completed. Participants will complete the T1 to T4 assessments following the schedule described for SAM-only participants.

###### MI Interventionist Training and Fidelity Assessment

The full-time study interventionist, an individual with a master’s degree in counseling, will receive refresher training from a supervisor who is a licensed clinical social worker regarding the principles of MI as they relate to substance use and the key components of the TRAC intervention through 12 hours of instruction and mock interviews. This supervisor will review the interventionist’s first TRAC case (or additional cases, if needed). Fidelity ratings will be conducted using a combination of the Motivational Interviewing Treatment Integrity (MITI) 4.2.1 coding system and adherence checklists to be developed by the study team to code for the presence of specific skills and exercises included in the TRAC protocol. MITI is an established structured behavioral coding system that serves as a treatment fidelity measure for clinical trials of MI [37,38]. It provides a global impression of empathy and MI spirit, a reflection-to-question ratio, and summary scores for MI components based on behavior count ratios of complex reflections, open questions, and MI-adherent utterances. The interventionist will be *certified* as able to deliver the protocol with fidelity. Certification will involve meeting adherence of at least 80% of TRAC-specific skills and exercises and the recommended MI competency threshold on at least 80% of sessions for a given case. Until the interventionist is certified, the supervisor will review entire session recordings weekly to code TRAC adherence, and a randomly selected 20-minute segment will be coded with MITI. Individual feedback and training will be provided weekly. Overall trial fidelity ratings (adherence to TRAC skills and exercises and MI competency) will be determined via review of an additional randomly selected 20% of interviews (10% from each site), in line with recommendations for RCTs of MI [39], including double coding by the supervisor and a research assistant, both of whom will be certified in MITI coding.

##### Data Collection Procedures

###### Overview

As this study is examining both short- and long-term effects and comparing them among the groups, the primary end point of the number of past-month drinking days will be assessed based on SAM results collected before T1, throughout the intervention, and before T3 and T4. Secondary end points include drinks per drinking day and binge drinking episodes. All other variables measured, including those related to HIV risk behaviors, will be used to conduct exploratory analyses. The sources of data for this RCT include SAM data (including surveys and breathalyzer results, discussed in detail previously), baseline and follow-up self-report measures (T1-T4), HIV and STI testing, app-based process measures, and qualitative interviews.

###### Baseline and Follow-Up Measures

An assessment battery will be administered to all participants at T1, T2, T3, and T4. This will include several measures of alcohol use as well as more exploratory variables, including alcohol cravings, alcohol consequences, the use of alcohol treatment services, HIV risk behaviors, and experiences of discrimination related to participants’ identity as sexual and gender minority individuals. Table 2 presents a description of the key study measures.

**Table 2:** Description of key study measures.

Construct and time		Measure	End point	Description
Drinking days, 2× daily		Breath alcohol content	Primary	BACtrack Mobile breathalyzer result sent via mobile app; measured 2× daily for 8 weeks during the intervention and for 1 month before T1^a^, T3^b^, and T4^c^; 2 negative readings per day=nondrinking day
Drinks per drinking day, daily		Self-reported alcohol use through app	Secondary	3 items: alcohol consumed (yes or no), number of drinks, and time spent drinking; measured daily during the intervention and for 1 month before T1, T3, and T4
Binge drinking episodes, daily		Self-reported alcohol use through app	Secondary	Occasions when participants had ≥5 drinks based on self-reported number of drinks; measured daily during the intervention and for 1 month before T1, T3, and T4
Alcohol withdrawal symptoms, daily		Single-item AWSC^d^ [40]	Exploratory	1 item: alcohol withdrawal symptom severity on a scale of 0 to 9; those scoring ≥2 are asked to complete the 17-item AWSC, which assesses the severity of symptoms; measured daily during intervention and for 1 month before T1, T3, and T4
Sexual activity, daily		Self-reported through app	Exploratory	6 items: past-day sexual activity, number of partners, partner type, partner HIV status, type of sexual activity, condomless sex, and PrEP^e^ use
Location characteristics, up to 2× daily		Self-reported through app	Exploratory	12 items assessing alcohol and substance use, sexual behaviors, peer use of alcohol or substances, and harassment or discrimination experienced
Alcohol use, T1, T2^f^, T3, and T4		PROMIS^g^ Short Form–Alcohol Use 7a [41]	Exploratory	7 items that assess general alcohol use and problem drinking in the past 30 days (eg, drank too much and drank heavily)
**Alcohol consequences**				
	T1, T2, T3, and T4	PROMIS Short Form–Alcohol Use–Negative Consequences 7a [41]	Exploratory	7 items that assess the negative consequences of using alcohol in the previous 30 days (eg, unreliability, social problems, and judgment)
	T1, T2, T3, and T4	PROMIS Short Form–Alcohol Use–Positive Consequences 7a [41]	Exploratory	7 items that assess the positive consequences of using alcohol in the previous 30 days (eg, self-esteem, confidence, and enjoyment)
**Alcohol expectancies**				
	T1, T2, T3, and T4	PROMIS Short Form–Alcohol Use–Negative Expectancies 7a [41]	Exploratory	7 items that assess the negative expectancies of alcohol use (eg, making bad decisions and behaving rudely)
	T1, T2, T3, and T4	PROMIS Short Form–Alcohol Use–Positive Expectancies 7a [41]	Exploratory	7 items that assess the positive expectancies of alcohol use (eg, improves mood and sociability)
Readiness to change drinking, T1, T2, T3, and T4		Readiness to Change Questionnaire [42]	Exploratory	12 items that assess the stage of change toward reducing alcohol use (precontemplation, contemplation, and action)
Drinking refusal self-efficacy, T1, T2, T3, and T4		Drinking Refusal Self-Efficacy Questionnaire–Revised [43]	Exploratory	19 items that assess individuals’ belief in their ability to resist alcohol, with 3 subscales: social pressure drinking, emotional relief drinking, and opportunistic drinking
Sexual risk behaviors, T1, T2, T3, and T4		National HIV Behavioral Surveillance System [44]	Exploratory	≥6 items: number of partners in previous 12 (T1) or 3 (T2-T4) months; partner type (main vs casual); partner HIV status; frequency of condomless sex (anal, vaginal, or oral), transactional sex, and concurrent sex and drug or alcohol use; and PrEP use
Condom attitudes, T1, T2, T3, and T4		Multidimensional Condom Attitudes Scale [45]	Exploratory	16 items that measure attitudes toward condoms across 7 factors: reliability, effectiveness, pleasure, identity stigma, embarrassment about purchase, negotiation, and action maintenance
Perceived HIV risk, T1, T2, T3, and T4		Perceived Risk of HIV Scale [46]	Exploratory	8 items that assess likelihood estimates, intuitive judgments, and salience of HIV risk based on previous behaviors
Discrimination due to sexual or gender identity, T1, T2, T3, and T4		Heterosexist Harassment, Rejection, and Discrimination Scale [47]	Exploratory	12 items that assess harassment, rejection, and family discrimination related to LGBTQ^h^ identities
Alcohol cravings, T1, T2, T3, and T4		Penn Alcohol Craving Scale [48]	Exploratory	5 items that assess alcohol-related cravings; higher scores indicate higher rates of alcohol cravings
Alcohol Use, T1, T2, T3, and T4		Alcohol Quantity Measures	Exploratory	7 items that assess alcohol use over the past 30 days

^a^T1: baseline assessment.

^b^T3: 6-month assessment.

^c^T4: 9-month assessment.

^d^AWSC: alcohol withdrawal symptom checklist.

^e^PrEP: pre-exposure prophylaxis.

^f^T2: 3-month assessment.

^g^PROMIS: Patient-Reported Outcomes Measurement Information System.

^h^LGBTQ: lesbian, gay, bisexual, transgender, and queer.

###### HIV and STI Testing

At T1 and T4, participants will receive HIV and 3-site gonorrhea and chlamydia testing. We will use myLAB Box, with whom we have worked successfully in prior research, to deliver home testing kits to participants. myLAB Box is a fully laboratory-certified company using College of American Pathologists– and Clinical Laboratory Improvement Amendments–certified testing organizations and a HIPAA (Health Insurance Portability and Accountability Act)–compliant web interface [49]. The home testing kits include written instructions for how to collect the samples; participants will complete a finger prick blood sample for the HIV test and collect swab and urine samples for the gonorrhea and chlamydia testing. The test kits will be mailed by the participants to myLAB Box using prepaid shipping boxes. Before completing testing, participants will complete a HIPAA release form giving permission for their results to be shared with the study team. In the case of a positive test, the participant will have the opportunity to complete a telehealth visit (free of charge) with one of myLAB Box’s providers to discuss medications and linkage to care. The research team will also follow up with the participant to ensure that they have been connected to care and, in the case of a positive HIV test at T1, to communicate that they are no longer eligible to participate in the study.

###### Process Measures

We will assess several process measures related to the study app, including the number of opened and completed daily surveys, the number of messages opened, the length of time messages stayed open, the number of completed breathalyzer readings and location-based surveys, the frequency of missing GPS data, and the number of reported app issues. For each participant completing monitoring, we will generate a weekly report of these metrics to address any ongoing technical issues in a timely manner and to encourage the completion of surveys and breathalyzer readings if they are nonadherent. For the TRAC intervention, the interventionist will complete fidelity checklists after each session. We will also assess session attendance, dropout rates, and time spent in sessions. At the end of the intervention, we will conduct an overall fidelity assessment to aid in the interpretation of the intervention results. Overall, these process measures will inform future dissemination and implementation efforts.

###### Qualitative Intervention Assessment

At the T2 assessment, we will conduct a semistructured qualitative interview to obtain feedback on the SAM-only, and SAM+ TRAC, and SAM + TRAC-ER conditions. As applicable according to their intervention group, the interview will address the effects of the monitoring of alcohol use, attitudes toward MI intervention content, the impact of the EMI messages on drinking and HIV risk behavior, the impressions of intervention relevance and effects, challenges encountered during the intervention, and suggestions for improvement.

###### Participant Training

Participants in each condition will be trained to navigate the app and complete tasks, including completing location-based and daily assessments, reading messages, and providing breathalyzer readings. They will also be instructed on data safety and confidentiality procedures and how to remotely wipe their mobile phone data if it is lost or stolen. Wallet-size cards with information from the training and text reminders to carry the mobile phone and breathalyzers will be provided. A lack of response will be followed up with texts and telephone calls to ascertain the reason for noncompliance. If participants report problems, we will complete video calls to troubleshoot and further train participants, if needed.

###### Methods to Decrease Attrition and Missing Data

During the first 3 months of the study, participants will receive daily reminders to complete SAM, which will include information about potential incentives. Individuals in the SAM+TRAC and SAM+TRAC-ER arms will have weekly contact with research staff during the TRAC sessions and will receive reminders about upcoming appointments. We will contact participants 1 month before their follow-up monitoring periods to remind them about upcoming tasks and to ensure that contact information is up to date. The use of a novel smartphone app and a mobile breathalyzer, which has been highly rated by participants in past studies, will also help to ensure continued engagement with, and enthusiasm for, the study.

##### Ethical Considerations

All procedures were approved by the University of Kentucky Institutional Review Board (79109), with Yale University relying on the University of Kentucky for human participant oversight. All responses of participants will be held in confidence and kept secure. Only the researchers involved in this study and those responsible for the research oversight will have access to the information that may identify the participants.

All participants will complete informed consent before beginning research activities. All consenting procedures will be completed remotely and electronically using the REDCap (Research Electronic Data Capture; Vanderbilt University) *eConsent* framework. First, participants will be given basic information about the study. If they indicate interest in learning more and meet eligibility criteria after completing a brief screening survey, a member of the research team will contact them via their preferred contact method to confirm eligibility, provide more details about the research procedures, and obtain informed consent.

The research team will review all aspects of the study, including specific research procedures, the length of time participants will be enrolled in the study, and the associated risks and benefits of participation (eg, benefiting from free alcohol reduction counseling, which may impact overall health, and the risks of emotional distress from completing sessions and assessments and of a loss of confidentiality). Contact information for study staff, the principal investigators, the University of Kentucky’s Office of Research Integrity, and Yale University’s Human Research Protection Program will be provided, and the participant will be encouraged to contact any of the relevant individuals if they have questions or concerns regarding this research or to discuss their rights as a research participant.

If a participant chooses to sign up for the study after this conversation with the research team, they will complete consent by providing an electronic signature in REDCap. A copy of the consent form will be provided to the participant electronically and retained in REDCap’s file repository for study records. By signing this form, participants agree to allow for future use, and sharing of, their deidentified study data with other researchers, without additional informed consent. No participant will begin research procedures without first providing signed informed consent.

To protect confidentiality, participants will be assigned a unique study ID number. Any identifying information will not be stored alongside survey responses and will be permanently deleted after the completion of data collection in accordance with the University of Kentucky Institutional Review Board policies.

The research team will take all precautions to prevent a loss of confidentiality, one of the primary risks of this study. Data collected and stored on mobile phones are inherently less secure than other storage methods; however, participants will be advised on how to protect data stored on mobile phones, and study staff will be trained on best practices for securing information on mobile phones.

Electronic data will be stored and managed in REDCap and will be accessible only to authorized study personnel. All data collected through the mobile app will be hosted on a secure private server at Yale University. myLAB Box uses a HIPAA-compliant web portal that encrypts all medical information, and all communications sent by the company will minimize personal details. Due to the sensitive nature of HIV and STI results, we will give the test results a unique ID in addition to the study ID and maintain them in a separate REDCap database without other identifying information. Access to these, and all other study data, will be permitted only to authorized personnel, who will be trained in the areas of ethics, clinical trials, confidentiality protection, and human participant protection.

Participants can earn up to US $825 (US $685 for monitoring tasks and US $140 for assessments). All participants will receive a US $1 compliance payment for each breathalyzer sample submitted within a 90-minute period. They will also receive a US $1 payment for submitting their daily morning surveys. Thus, those who complete all monitoring tasks (all breathalyzer readings and daily self-reports) during the whole study (4 weeks before T1, 8 weeks during the intervention, and 4 weeks before T3 and T4) will receive US $685. Participants will also receive US $35 for completing the T1 assessment (US $20 for the questionnaire and US $15 for HIV and STI testing), US $25 for T2, US $30 for T3, and US $50 for T4. Escalating payments will be used to discourage study attrition.

While the compensation amount is relatively high, it is for activities completed across a full 9 months of participation. Total time spent on study activities ranges from 45 hours (SAM-only participants) to 50 hours (SAM+TRAC and SAM+TRAC-ER participants) when considering daily monitoring, assessments, time spent on screening and enrollment, and time spent in MI sessions. This results in an average compensation of US $16-$18 per hour in exchange for participation in study activities.

We will pay participants with reloadable debit cards, administered using OnCore clinical trial software (Advarra). Funds will be added to these cards as payments are earned, which then become immediately available. This system will allow for frequent payments without the burden of office visits or the delays from mailing checks.

##### Data Analysis Plan

###### Analysis of Alcohol Use Outcomes

Descriptive statistics for breathalyzer reading data will be summarized overall, by group (SAM only, SAM+TRAC, and SAM+TRAC-ER) and across time periods (T1-T4). The primary outcome is the number of drinking days in the past month, which is defined as the days on which individuals did not report 2 negative breathalyzer results. The rate of drinking (incidence of drinking) is the number of drinking days observed within the past month. The secondary outcomes include the number of drinks per drinking day, the number of days with binge drinking episodes, and the number of days for which HIV risk behaviors occur. All these outcomes are count data. The goal of this analysis is 2-fold: first, short-term analysis at the end of the 2-month intervention period, and, second, long-term analysis at the end of 9 months. We will fit a generalized linear model [50], with a general formulation for count data as follows:

g(E(y_i_)) = g(μ_i_) = x'_i_β (1)

where *y_i_* is the outcome variable for (*i*=1,2,3,...,*n*), *n* is the total number of observations, *μ*_i_=*E*(*y_i_*) is the expected count, *g* is the link function (a mathematical function connecting the counts with the explanatory variables), *β* is a vector of regression parameters to be estimated, and *x_i_* is a vector of independent covariates to be included in the model. These covariates include group, time, age (or age group), gender identity, sexual orientation, and site.

###### Short-Term Outcome Analyses

After 3 months, we will fit the aforementioned model to the data and compare the groups at T1 and T2 while adjusting for the covariates. The PROC GENMOD procedure in SAS (SAS Institute Inc) will be implemented to estimate the parameters of the model, where we specify a log-link function, Poisson distribution for the count data, an offset (which is a function of the total exposure), and the covariates. We will address overdispersion using negative binomial distribution. Overdispersion occurs when the expected count for the outcome variable is not the same as its variance. The generalized estimating equation procedure will be used to account for correlation induced by the repeated observations at T2.

###### Long-Term Outcome Analyses

At 9 months, the time variable will include observations at T1 to T4, and the procedure implemented for the short-term analysis will be repeated with the inclusion of additional covariates in the form of interaction terms among the groups and follow-up times (T1-T4). The interaction effect is central to the comparison of the groups over the study period. Generalized estimating equation fit criteria and quasi-likelihood under the independence model criterion value will be used to determine the adequacy of model fit. This modeling framework facilitates the easy computation of hypothesis tests and CIs for effects, which will form the basis of our inferential strategy in this project. Specifically, contrast statements will be used to test specific subgroup comparisons. Multiple imputation procedure will be implemented whenever necessary for missing data points. SAS (version 9.4 or higher) will be used for all analyses, and a standard 5% significance level will be used for all statistical tests.

###### Addressing Biological Variables

As this project is focused on SMMT individuals, rather than assessing the effects of biological sex, we will screen for the effects of gender identity and sexual orientation in the aforementioned modeling framework. This is accomplished by introducing indicator variables for these subgroups into the analyses and assessing statistical and practical significance. The stratified randomization procedure should prevent systematic differences at baseline among the groups. We will check for statistically significant differences after randomization and adjust for such differences in the analysis in the rare event that they manifest.

###### Qualitative Data Analysis

Audio files of postintervention interviews will be transcribed and imported into NVivo software (Lumivero). Each transcript will be examined and coded using a grounded thematic approach [51], drawing on observed themes regarding intervention attitudes, experiences, and effects. The research team will develop the coding scheme based on an initial review of the transcripts; next, 2 coders will independently code a random sample of 10% of the transcripts. Interrater reliability will be assessed, and the coding scheme will be refined if reliability is not acceptable (acceptable interrater agreement: >90%). Once interrater agreement is achieved, the coders will code the remaining transcripts and collect representative quotations.

###### Gender-Inclusive and Gender-Specific Approach to Analysis

As an analytic framing to examine inequities in alcohol behaviors within SMMT populations, we will use gender-inclusive and gender-specific approaches throughout the study’s quantitative and qualitative analyses, following guidelines from Restar et al [52]. Given that there is epidemiological evidence that transgender and cisgender populations’ health outcomes and behaviors, such as alcohol use, are dissimilar to each other and that the drivers of these inequities are contextually uniquely different [6,53-55], applying this approach to our analysis is appropriate. This approach allows our analytical modeling to examine and discern any potential shared versus differential intervention experiences and outcomes across gender groups (ie, between cisgender and transgender participants and within gender subgroups of transgender participants). On the basis of the findings, it will also allow us to provide future intervention recommendations for either the entire sample (ie, gender inclusive) or specific to each gender group (ie, gender specific).

###### Scientific Rigor

The proposed study uses a rigorous randomized comparative effectiveness trial design to assess intervention effects on alcohol use outcomes. The following characteristics add to the rigor of the study: (1) the use of a SAM-only control group so that all groups can be compared using daily-level alcohol use data, (2) the incorporation of breathalyzers to enhance the validity of self-reported alcohol use, (3) the inclusion of follow-up self-report and SAM data to assess long-term outcomes, (4) recruitment strategies that use in-person and web-based methods to enhance the diversity of the sample, (5) the inclusion of a dedicated biostatistician to provide rigorous outcome analyses, (6) support from app programmers to customize the app and address technical issues, and (7) the inclusion of a licensed social worker to ensure high-quality MI training and fidelity.

## Results

The study is part of a 5-year National Institutes of Health research project that was funded in August 2022 by the National Institute on Alcohol Abuse and Alcoholism. The first 1.5 years of the study will be dedicated to planning and development activities, including formative research, app design and testing, and message design and testing. The subsequent 3.5 years will see the study complete participant recruitment, data collection, analyses, report writing, and dissemination. We expect to complete all study data collection in or before January 2027.

As of February 2024, we have published 1 manuscript, a systematic review exploring interventions for addressing alcohol use and sexual HIV risk–related behaviors [16]. We have also completed 2 rounds of focus groups to refine the intervention content and harm reduction messages delivered in TRAC-ER. Overall, 34 participants were enrolled in the first round of focus groups; however, 5 were excluded after further review showed they did not meet the eligibility criteria. This resulted in a final sample of 29 individuals for the first round of focus groups. We enrolled 22 participants for the second round of focus groups. First, we conducted separate focus groups with participants of different gender identities (sexual minority cisgender men, nonbinary and gender nonconforming participants, transgender men, and transgender women) to obtain feedback on harm reduction content added to the intervention manual and to help us generate initial harm reduction messaging. Next, we conducted an additional blended focus group to help us further refine the messages and develop our final *library* of messages that will be programmed into the app. A manuscript describing this process and the results is forthcoming.

## Discussion

### Summary

This manuscript describes the protocol for a 3-arm RCT evaluating the efficacy of a mobile-based EMI for reducing alcohol use and HIV risk behaviors among emerging adult SMMT individuals (N=405). We will compare a monitoring-only control to TRAC, a mobile-based MI intervention, and to TRAC-ER, an intervention combining TRAC with in-the-moment messaging to prevent hazardous alcohol use and reduce harm when participants visit risky locations. Mobile breathalyzers and surveys will be used to collect frequent data on alcohol and HIV risk behaviors. We will assess outcomes immediately before and after the intervention, as well as at 6-month and 9-month follow-ups. The primary outcome is the number of drinking days. Formative development work for this RCT has been completed, with the trial to begin recruiting soon (as of February 2024).

This study will make significant contributions to the literature on alcohol use interventions for SMMT populations. It uses an innovative precision health approach that draws upon the frequent use of smartphone technologies by emerging adult populations and the high acceptability of mobile breathalyzers. Despite the proliferation of mobile devices in society, the field of EMIs remains understudied, with very few RCTs being conducted related to alcohol use. In addition, EMIs have shown initial promise in being paired with MI approaches [56-58]; therefore, the proposed study would help to add to the fledgling evidence base regarding the effectiveness of this approach. The use of mobile technology and remote delivery of the tested interventions means that once the ideal approach is identified in terms of efficacy, the intervention will be easily scalable to individuals across wide geographic areas.

Our study has promising strengths, but it must be considered in light of several limitations. While the use of ecological momentary assessment (EMA) provides advantageous opportunities for real-time trigger location data and convenience reporting through smartphones, prior EMA studies highlight the potential exhaustion that participants may experience, which may reduce the level of participation. This study may further explore convenient assessment windows to diminish the intensity of EMA sampling. In addition, as previously mentioned, SMMT communities experience unique sociostructural challenges, such as unemployment, homelessness, forced displacement, and community violence [59]. These systems of power may operate dynamically and render difficulties in participating fully and equitably in hazardous alcohol use prevention programs [60]. Finally, the real-time tracking of alcohol use behaviors of participants may give rise to reporting bias. In the context of alcohol-related stigma, social desirability biases may subject participants to aligning their responses to the expectations of societal norms around alcohol consumption; however, the use of breathalyzers will help to protect against self-report bias.

Prior studies have also reported concerns for temporal bias, where participants were reluctant to complete location- and time-based–triggered EMA surveys due to the inconvenient times at which the surveys were sent. Similarly, EMA studies related to substance use have mentioned challenges with the time frame for EMA sampling [61]. In this study, EMA assessments will occur between 11 AM and 11 PM to avoid disturbing participants. However, participants in prior studies have discussed that these hours may not be representative of the time frames when drinking occurs because alcohol use may occur later at night, along with variations in mood and social activities. Thus, we may explore the potential options for personalized hours of EMA sampling for participants. Breathalyzer device loss may also pose a challenge. However, unlike prior EMA studies that distributed mobile phones and experienced challenges with device loss [62], our study enables participants to use their existing smartphones to use the app, which will reduce instances of device loss or misplacement.

We plan to follow this study with dissemination and implementation research that takes the most effective approach identified here and distributes it to a wider population of SMMT individuals. Overall, by reducing hazardous alcohol use among emerging adult SMMT individuals, we hope to reduce the rates of alcohol-associated HIV transmission among this population considered to be at high risk.

### Conclusions

This protocol describes an RCT of an innovative intervention for reducing alcohol use and HIV risk among SMMT individuals. This study will provide evidence about the relative efficacy of using a smartphone-delivered MI intervention and real-time messaging to address triggers for hazardous alcohol use and sexual risk behaviors. The study has potential implications for advancing science and improving public health for a subpopulation that is disproportionately stigmatized and disconnected from prevention research and services.

## Appendix

Multimedia Appendix 1 Peer review report from the National Institutes of Health.

## Abbreviations

AUD: alcohol use disorder
AUDIT-C: Alcohol Use Disorders Identification Test–Concise
EMA: ecological momentary assessment
EMI: ecological momentary intervention
HIPAA: Health Insurance Portability and Accountability Act
MI: motivational interviewing
MITI: Motivational Interviewing Treatment Integrity
PrEP: pre-exposure prophylaxis
RCT: randomized controlled trial
REDCap: Research Electronic Data Capture
SAM: smartphone-based alcohol monitoring
SMMT: sexual minority cisgender men and transgender
STI: sexually transmitted infection
TRAC: Tracking and Reducing Alcohol Consumption
TRAC-ER: Tracking and Reducing Alcohol Consumption and Environmental Risk

## Data Availability

The data sets generated and analyzed during this study are available in the National Institute of Mental Health Data Archive repository (ID C4476) [63].
