# Supplementary material for: Combined Motivational Interviewing and Ecological Momentary Intervention to Reduce Hazardous Alcohol Use Among Sexual Minority Cisgender Men and Transgender Individuals: Protocol for a Randomized Controlled Trial
Source: JMIR Res Protoc. 2024 Apr 5;13:e55166. doi: 10.2196/55166 (PMC11031694; doi:10.2196/55166)
Supplement: Multimedia Appendix 1 [file resprot_v13i1e55166_app1.pdf]

1R01AA030487-01 LAUCKNER, CAROLYN

RESUME AND SUMMARY OF DISCUSSION: This is a new multi-PI application submitted in response to RFA AA 21-016 HIV Prevention and Alcohol (R01 Clinical Trials Optional). The applicants propose a randomized controlled trial to evaluate an enhanced motivational interviewing intervention that combines smartphones and mobile breathalyzers to provide real-time messaging to control drinking triggers when individuals visit locations associated with risky alcohol use. The strengths of the application include a strong team of investigators with complementary expertise, and an innovative 3-arm randomized controlled trial design comparing TRAC – a remotely delivered motivational interviewing intervention and GeoTRAC that TRAC with an ecological momentary intervention to deliver drinking reduction text messages when individuals visit risky locations. Some of the weaknesses noted include concerns about feasibility/efficacy of geofencing, feasibility/timeline of GPS-EMA software development, and inadequate discussion of scale and dissemination plans. Overall, the strengths outweigh weaknesses and there was a strong enthusiasm for this application.

DESCRIPTION (provided by applicant): Sexual and gender minority individuals are more likely to report hazardous drinking and have alcohol use disorders (AUDs) compared to cisgender and heterosexual people. Emerging adult (aged 18-34) sexual and gender minorities are at especially high risk for AUDs compared to older adults, with up to 44% meeting AUD criteria. These trends are concerning, as studies have found that alcohol use increases HIV risk behaviors, and HIV disproportionately affects emerging adult sexual minority male and transgender (SMMT) individuals. Thus, it is essential to test interventions for reducing alcohol use among SMMT individuals as a means of preventing HIV among this vulnerable population. One potential evidence-based method of reducing alcohol use is motivational interviewing, which incorporates a goal-oriented approach for changing behavior and has been shown to be effective among various populations in prior literature and the investigators' pilot research. However, there are opportunities to use technology to extend the impact of this approach by delivering messaging to prevent risk behaviors in real time (i.e., outside of intervention sessions). The proposed study will conduct a 3-arm randomized controlled trial that assesses the efficacy of two interventions as compared to a control: 1) An 8-week motivational interviewing intervention (TRAC) delivered remotely via cell phones and 2) TRAC combined with an ecological momentary intervention designed to deliver messages reinforcing drinking reduction strategies discussed during TRAC sessions when individuals visit risky locations (GeoTRAC). All participants will also complete monitoring of alcohol use and sexual behavior by completing daily surveys and twice-daily mobile breathalyzer readings. GeoTRAC will use an existing app developed in the investigators' ongoing research that uses GPS tracking to determine when individuals visit "risky" locations. Participants will receive messages upon arrival at these locations reminding them of strategies to address triggers for drinking, and will also complete mobile surveys and breathalyzer readings when they leave these locations to determine what alcohol and/or risk behaviors they engaged in. If they reported drinking, they will receive harm reduction messaging to encourage them to avoid subsequent risk behavior (e.g., unprotected sex). Breathalyzer results and daily self-reports will be used to assess the primary and secondary outcomes of drinking days, drinks/drinking day, binge drinking episodes, and HIV risk behaviors, and additional assessments at baseline, 8 weeks, 6 months, and 12 months will evaluate exploratory long-term outcomes. Participants will be recruited from Kentucky and Connecticut through community-based recruitment and health clinics that serve SMMT individuals. Overall, there is a strong need to determine optimal methods of reducing alcohol use among emerging adult SMMT individuals given its relationship to increased HIV risk behaviors. The proposed work will draw upon the high technological literacy of emerging adults by using mobile phones and will test an innovative precision medicine approach that offers comprehensive support in reducing hazardous alcohol consumption.

PUBLIC HEALTH RELEVANCE: Emerging adult sexual minority male and transgender individuals are more likely to report hazardous drinking and to have alcohol use disorders compared to those who are

cisgender and heterosexual. This is concerning, as alcohol use contributes to HIV risk and HIV disproportionately affects this population. Through a randomized controlled trial, the proposed research will evaluate an enhanced motivational interviewing intervention that utilizes smartphones and mobile breathalyzers to provide real-time messaging that addresses triggers for drinking when individuals visit locations associated with risky alcohol use.

CRITIQUE 1
Significance: 3
Investigator(s): 3
Innovation: 2
Approach: 6
Environment: 1

Overall Impact: This proposal aims to conduct a 3-arm randomized controlled trial that assesses the efficacy of two interventions relative to control in reducing alcohol use and HIV-related sexual risk behavior among sexual minority men and transgender people. The two active interventions are: 1) an 8-week motivational interviewing intervention (TRAC) delivered remotely, and 2) TRAC combined with an ecological momentary intervention that will deliver drinking reduction text messages when individuals visit risky locations (GeoTRAC). The control condition will be EMA completion only (i.e., daily surveys and twice daily mobile breathalyzer). Breathalyzer results and daily self-reports will be used to assess the primary and secondary outcomes of drinking days, drinks per drinking day, binge drinking episodes, and HIV risk behaviors at 8 weeks, 6 months, and 12 months post-intervention. The proposal is seen as significant, the investigative team is strong, and there are some interesting points of innovation (e.g., geographically triggered risk reduction messaging). However, there are also a number of issues that dampen my enthusiasm. Major concerns include the grouping together of sexual minority men and transgender people throughout the proposal (without consideration of these groups' unique needs), lack of clarity in the description of the risk reduction text messages, and issues related to recruitment.

1. Significance:

Strengths

- Sexual minority male and transgender emerging adults have high rates of hazardous drinking and alcohol use disorders, and this is particularly pronounced among emerging adults.
- These groups are also disproportionately impacted by HIV.
- Alcohol use is linked to various indicators of risk for HIV, so reducing alcohol use may also reduce risk for HIV infection.
- Motivational Interviewing is an effective strategy for reducing alcohol use and HIV-related risk behaviors among MSM.

Weaknesses

- Much of the data presented to support this study are from samples of sexual minority males, and there are times when these data are used as citations to support statements about specific health behaviors/risks of transgender people. Overall, more attention is needed to the unique/common experiences of transgender people in order to effectively include them in the study.

2. Investigator(s):

Strengths

- PI Lauckner is strong early career investigator with an impressive portfolio of funded research and existing collaborations with the other MPI.
- PI Kershaw has extensive experience in HIV and alcohol use among MSM.
- This proposal has a strong biostatistician.
- Co-I Fallin-Bennet has nice complementary experience working with SGM patients in healthcare settings.
- The presence of an endocrinologist who works with transgender patients is an asset.

Weaknesses

- While the team has experience with transgender people in a clinical context, there is no apparent expertise in research with transgender people, particularly research on alcohol use and HIV.
- The MPI plan doesn't say much about how the PIs roles differ, except that each will run operations at their own site. I'd like to see more detail about the differing areas of expertise and/or different roles each PI will play.

3. Innovation:

Strengths

- The ability to provide risk reduction messages in the moment when participants enter venues they've previously designated as "risky" is novel and could have high impact.
- The use of a mobile breathalyzer to get an objective measure of alcohol use is innovative.

Weaknesses

- None noted

4. Approach:

Strengths

- The team presents strong preliminary data on TRAC with convincing interim analyses.
- The inclusion of risk reduction messages that are triggered by geographical factors is strong.
- The 3-arm RCT is well-described and robust.

Weaknesses

- One of my biggest concerns is the grouping together of sexual minority men and transgender people throughout the proposal and study design. Transgender people have unique experiences that likely influence their drinking and sexual behavior. Will these be addressed? If so, how? It is also not evident whether the team has experience recruiting transgender people into research from these clinical settings.
- The investigators present a conceptual model for their intervention that includes numerous antecedents (e.g., stress, stigma), but it is not clear as to how/if these antecedents are integrated into the intervention.
- I have some concerns about the recruitment plan. Based on the recruitment strategy, there will be major differences between sites in the proportion of transgender people and sexual minority men. And there will also be major differences between sites in the specific recruitment

strategies being used. Thus, it will not be clear if observed differences are due to gender identity, recruitment method, or state/region.

- Another major issue is that there will not be an inclusion criterion for HIV risk. There is high variability between individuals in HIV risk, even among drinkers. Thus, secondary analyses on HIV risk behavior will likely be underpowered. Further, it won't be clear if participants are even having sex with men at the time of enrollment (e.g., bisexual individuals, transgender people who don't have sex with men), and these people will be at very low risk for HIV.
- There is some lack of clarity in the content of the text messages. How are the messages being generated? How is the team selecting what messages to send at different locations? How will the team handle situations like drinking at home (will participants get a notification every time they walk in their door)? Will the messages be tailored to the unique experiences of transgender people and sexual minority men, or will they be the same messages?
- The HIV-related outcomes lack precision, and there are no descriptions of the HIV-related analyses. For example, sex without a condom may not always be problematic (e.g., if the participant is in a seroconcordant monogamous relationship, the participant is on PrEP).
- How might incentivized participant referral influence the integrity of the RCT?

5. Environment:

Strengths

- The infrastructures at University of Kentucky and Yale are very strong and will support the proposed project.
- The Center for Health Equity Transformation and the CTSA at Kentucky are strong additional support.
- The Center for Interdisciplinary Research on AIDS at Yale is a strong environment for this research.

Weaknesses

- None noted.

Study Timeline:

Strengths

- The timeline is detailed and appropriate.

Weaknesses

- None noted.

Protections for Human Subjects:

Acceptable Risks and/or Adequate Protections

- Risks are minimal and protections acceptable.

Data and Safety Monitoring Plan (Applicable for Clinical Trials Only):

Acceptable

- o DSMP is detailed and appropriate.

Inclusion Plans:

- Sex/Gender: Distribution justified scientifically.
- Race/Ethnicity: Distribution justified scientifically.
- For NIH-Defined Phase III trials, Plans for valid design and analysis: Not applicable.
- Inclusion/Exclusion Based on Age: Distribution justified scientifically.
- Distribution justified scientifically across factors.

Resource Sharing Plans:

- Acceptable

Budget and Period of Support:

- Recommend as Requested

CRITIQUE 2

Significance: 2
Investigator(s): 1
Innovation: 1
Approach: 4
Environment: 1

Overall Impact: This project aims to conduct a trial to evaluate motivational interviewing with smartphone-based alcohol monitoring (TRAC/SAM) as compared to an enhanced version of TRAC/SAM that uses an ecological momentary intervention to trigger alcohol messages based on physical location. The hypothesis is that the enhanced intervention will lead to a larger reduction in number of drinking days than TRAC/SAM or SAM alone by offering more precise messaging and support. The team is excellent, the research design is rigorous, the concept is innovative, and the proposal builds on an excellent set of preliminary data. There are some concerns about how geofencing will work, software development plans and whether this is feasible in six months, and the potential for response fatigue with twice daily breathalyzers/surveys and how this will be accounted for.

1. Significance:

Strengths

- SGM experience high rates of hazardous drinking, which in turn increases the risk for HIV.
- Motivational interviewing could benefit SGM who drink especially if combined with an ecological momentary intervention.

Weaknesses

- The lack of evidence on MI approaches for SGM may warrant a more preliminary study before full-scale efficacy trial. Also questions are raised about whether MI is effective at changing

shorter term outcomes, somewhat throwing into question the TRAC intervention which is based on MI.

2. Investigator(s):

Strengths

- MPI Lauckner is a productive early-stage researcher with sufficient experience in developing and testing interventions using MI with breathalyzers. MPI Kershaw is a senior researcher and adds complementary leadership around EMI interventions targeting alcohol use.
- Dr. Fallin-Bennet adds clinical expertise in this study population; Dr. Boyd will facilitate MI trainings; Dr. Hansen also has experience in the study population and in brief alcohol interventions.

Weaknesses

- None noted

3. Innovation:

Strengths

- Employing EMI using activity space assessment to reduce alcohol use is novel.
- The intervention will be fully-remote with potential for future scale-up, if efficacious, and has the potential to reduce disparities in access to alcohol services in this vulnerable population.

Weaknesses

- Smartphone apps to reduce alcohol use are not necessarily novel.

4. Approach:

Strengths

- The combination of Lauckner's research on TRAC and Kershaw's work on the activity space assessments may be synergistic. The prior work suggests both components are very promising and may have a larger impact when combined.
- Activity space assessments to identify risky locations and trigger alcohol messages may offer more personalized alcohol support by responding in the moment.
- MI training of facilitators and fidelity assessment plans are rigorous. It appears that facilitators may also receive their MITI certificate prior to the start. This is a plus.

Weaknesses

- The study could benefit from a stronger theoretical grounded in how components involving MI, monitoring, and location-based messaging will lead to the anticipated behavior changes.
- While the study population has access to smartphones, their technical literacy is not well described, and it is not clear how support for technical difficulties will be provided for less technically-advanced participants.
- Unclear if prior work also offered incentivizes for completing breathalyzers and daily surveys. This would offer support for the proposed strategy.
- A more major concern is the extent of the software development for the app, which often takes significantly longer than expected (only 6 months are budgeted). It is not clear what the app

currently does and what will be added or modified for the proposed study. These plans should be more detailed to demonstrate feasibility.

- The list of “risky places” will be pre-determined qualitatively and then compared against GPS location, however, it is not clear how messages will be handled for people who mostly drink at home or at a friend’s house. This could lead to fatigue, non-response, and reduced impact on behavior if messages are sent frequently.
- More detail could be provided on how participant GPS location will be compared against coordinates of risky locations and the range of error (is this off-the-shelf software or will this functionality be developed?).
- It is not clear how assessments will be administered (e.g., phone/online/CASI) and whether this approach has been successful in the past.
- Incentives will be used to maximize breathalyzer and survey responses, however, with 420 tasks per participant, response fatigue may still affect response rates and quality of data provided. There are no data to suggest what responses rates may be with this approach or plans to control for these issues in the analysis.

5. Environment:

Strengths

- Resources and infrastructure at University of Kentucky, Yale, and Ohio State University are excellent.

Weaknesses

- None noted

Study Timeline:

Strengths

- None noted

Weaknesses

- Only six months are planned for software development to update the app, which seems very short.

Protections for Human Subjects:

Acceptable Risks and/or Adequate Protections

Data and Safety Monitoring Plan (Applicable for Clinical Trials Only):

- Acceptable

Inclusion Plans:

- Sex/Gender: Distribution justified scientifically.
- Race/Ethnicity: Distribution justified scientifically.
- For NIH-Defined Phase III trials, Plans for valid design and analysis: Not applicable.
- Inclusion/Exclusion Based on Age: Distribution justified scientifically.

Budget and Period of Support

- Recommended as Requested

CRITIQUE 3

Significance: 2
Investigator(s): 2
Innovation: 2
Approach: 4
Environment: 1

Overall Impact: The proposed study focuses on a critical population: emerging adult sexual minority male and transgender individuals are disproportionately burdened by hazardous drinking and alcohol use disorders, which is concerning as alcohol use contributes to HIV risk and HIV disproportionately affects this population. The investigators propose a randomized controlled trial to evaluate an enhanced motivational interviewing intervention that utilizes smartphones and mobile breathalyzers to provide real-time messaging that addresses triggers for drinking when individuals visit locations associated with risky alcohol use. Overall, the reviewer expresses strong enthusiasm for this project, with some minor, addressable, concerns, mainly related to the lack of discussion about implementation science or focus on understanding further scale up or dissemination of the intervention approaches being evaluated. It is also not clear how the investigators will assess and account for treatment-seeking among participants.

1. Significance:

Strengths

- The target population of the proposed study, sexual minority male and transgender emerging adults frequently engage in hazardous drinking and have high rates of alcohol use disorders, which contributes to risk of HIV transmission.
- Ecological momentary interventions, when paired with an MI intervention, could provide real-time support in addressing triggers for drinking while also reinforcing content discussed during MI sessions.

Weaknesses

- No mention of implementation science or focus on understanding further scale up or dissemination of the intervention approaches being evaluated.

2. Investigator(s):

Strengths

- study team has experience in conducting MI interventions, working with SMMT populations in research and clinical settings, and using smartphones to collect substance use data.
- The planned research builds on ongoing grant-funded studies led by the investigators and represents an innovative step forward in their programs of research and in the literature regarding alcohol use interventions for SMMT individuals.

Weaknesses

- Not clear evidence of previous or ongoing collaborations among the study team, especially in the proposed study locations.

3. Innovation:

Strengths

- Assesses and addresses the role of geographic context.
- Leverages smartphone technologies.
- Objectively assess alcohol use with breathalyzer technology.
- Individually tailored intervention content.

Weaknesses

- None noted

4. Approach:

Strengths

- Proposed study builds off of promising preliminary studies being conducted by the investigative team.
- it is essential to test interventions for reducing alcohol use among SMMT individuals as a means of preventing HIV among this vulnerable population.
- Technology can be used to extend the impact of MI by delivering messaging to prevent risk behaviors in real time.

Weaknesses

- No mention of implementation science or focus on understanding further scale up or dissemination of the intervention approaches being evaluated.
- It is unclear how investigators will address who do not want to (1) reduce their drinking and/or (2) participate in the intervention (e.g., prefer different treatment approaches, among those randomized into different intervention arms)?

5. Environment:

Strengths

- All investigators' institutions are strong with adequate resources to successfully carry out the proposed project.

Weaknesses

- None noted

Study Timeline:

Strengths

- Appropriate study timeline.

Weaknesses

- None noted

Protections for Human Subjects

Acceptable Risks and/or Adequate Protections

- Acceptable

Data and Safety Monitoring Plan (Applicable for Clinical Trials Only):

- Acceptable

Inclusion Plans:

- Sex/Gender: Distribution justified scientifically.
- Race/Ethnicity: Distribution justified scientifically.
- For NIH-Defined Phase III trials, Plans for valid design and analysis: Scientifically acceptable.
- Inclusion/Exclusion Based on Age: Distribution justified scientifically.
- Acceptable

Resource Sharing Plans:

- Acceptable

Budget and Period of Support:

Recommended budget modifications or possible overlap identified:

- Acceptable

THE FOLLOWING SECTIONS WERE PREPARED BY THE SCIENTIFIC REVIEW OFFICER TO SUMMARIZE THE OUTCOME OF DISCUSSIONS OF THE REVIEW COMMITTEE, OR REVIEWERS' WRITTEN CRITIQUES, ON THE FOLLOWING ISSUES:

PROTECTION OF HUMAN SUBJECTS: ACCEPTABLE

INCLUSION OF WOMEN PLAN: ACCEPTABLE

INCLUSION OF MINORITIES PLAN: ACCEPTABLE

INCLUSION ACROSS THE LIFESPAN: ACCEPTABLE

COMMITTEE BUDGET RECOMMENDATIONS: The budget was recommended as requested.

Footnotes for 1 R01 AA030487-01; PI Name: Lauckner, Carolyn

NIH has modified its policy regarding the receipt of resubmissions (amended applications).See Guide Notice NOT-OD-18-197 at https://grants.nih.gov/grants/guide/notice-files/NOT-OD-18-197.html. The impact/priority score is calculated after discussion of an application by averaging the overall scores (1-9) given by all voting reviewers on the committee and

multiplying by 10. The criterion scores are submitted prior to the meeting by the individual reviewers assigned to an application, and are not discussed specifically at the review meeting or calculated into the overall impact score. Some applications also receive a percentile ranking. For details on the review process, see http://grants.nih.gov/grants/peer_review_process.htm#scoring.

Response to Reviews: Application # 1R01AA030487-01 (mPIs: Lauckner & Kershaw)

Our application had the benefit of a rigorous and favorable scientific peer review. We are pleased that the committee was highly enthusiastic about the innovation of the proposal, and that they felt the investigative team was strong with complementary expertise. This letter serves to further strengthen the application by responding positively to the few minor weaknesses raised by reviewers that did not damper the fact that the committee's overall enthusiasm for the application project remained high. Below are the main comments from reviewers and how we will modify our proposal to address these comments.

Justification for the inclusion of both sexual minority men (SMM) and transgender (trans) individuals, and discussion of how the unique experiences of trans people will be integrated in the study design.

Although there is considerably less research on trans individuals compared to SMM on alcohol use and HIV, the research available has shown: 1) high rates of alcohol use problems and alcohol use disorders for trans compared to cisgender populations,[1,2] and 2) the link between increased HIV risk behavior and both overall alcohol use and event-level alcohol use for trans individuals. [1,3] Further, the few motivational interviewing (MI) interventions that have been conducted with trans individuals have shown effectiveness on alcohol and substance use reduction, demonstrating promise for MI as a tool to reduce alcohol use among trans individuals.[4] This suggests that trans individuals are an ideal population for an alcohol and HIV risk reduction intervention using MI.

Further, reviewers wanted justification for combining SMM and trans individuals in the intervention given the differences between these populations. We will address the unique experiences and needs of SMM and trans individuals by: (a) using MI as the primary intervention component for both TRAC and GeoTRAC, a therapeutic technique that has been used successfully for both SMM and trans individuals[4] and which allows us to tailor intervention components based on the experiences, triggers, and context of the individual. Because MI uses an individualized approach, we will be able to deliver an intervention that is specific to the needs of SMM **and** trans individuals; (b) developing the place-based messaging used in GeoTRAC using triggers identified through MI sessions, which further facilitates tailored messaging and accounts for the specific experiences and needs of both SMM and trans individuals; (c) utilizing separate focus groups conducted with SMM and trans individuals (2 SMM and 2 trans sessions) to develop the harm-reduction messages (which are sent to GeoTRAC participants who test positive for alcohol using the breathalyzer), which will allow us to select messages that resonate the most for each subgroup; and (d) assessing for possible moderating effects by sexual and gender identity groups to determine if there are differential effects of the intervention by subgroup.

Finally, reviewers felt the team would be strengthened by adding a behavioral researcher with expertise in HIV and alcohol use among trans individuals to complement our strong clinical expertise in trans health. In response, we added Dr. Arjee Restar to our study team as a co-Investigator. Dr. Restar is an Assistant Professor in Epidemiology at the University of Washington and has over 25 publications related to trans populations, with specific expertise in HIV prevention, mental health, and substance use. [5-8] She will provide expertise in HIV and substance use among trans individuals and provide consultation on study and intervention design, recruitment techniques of trans individuals, and measurement and assessment.

More clarification on recruitment strategies and inclusion criteria.

Reviewers wanted clarification on how we would address differences in population characteristics (number of SMM/trans people) and recruitment strategies between the two sites. To address potential site effects, randomization will be stratified by site and SMM/trans subgroup, reducing the likelihood of confounding across conditions.

Reviewers also felt that because we did not include HIV risk level as inclusion criteria that we may be underpowered to detect differences on our secondary HIV-risk outcomes. To address this, we will include the CDC's PrEP eligibility criteria (as an indicator of HIV risk) as inclusion criteria. Individuals will be eligible if they have had anal or vaginal sex in the past 6 months *and* have at least one of the following characteristics: a sexual partner with HIV; inconsistent condom/PrEP use; or a sexually transmitted disease diagnosis in the past 6 months. This addition to our criteria will ensure participants have both high levels of alcohol and HIV risk.

More information on the feasibility of app development and testing

Reviewers were concerned about the feasibility of modifying our current app as needed for this study in the proposed 6 month timeline. The proposed app shares most of the same functionality as the apps used in our current RENEW and MVMNT studies (R01HD092185 and R01AA025954). Table 1 shows the overlapping functionality of the RENEW/MVMNT apps and GeoTRAC, with new functions bolded. The majority of the app functions are shared between RENEW/ MVMNT apps and GeoTRAC, suggesting modifications for GeoTRAC

will be more a refinement of functionalities than widespread development of new functions. Further, we recently modified the current app for an F31 to create an EMI intervention that delivers place-based messages (very similar to what we are proposing for GeoTRAC) for individuals in substance use treatment, and a beta version was created in <1 month. Given the overlap in functionality between the RENEW/ MVMNT apps and the proposed GeoTRAC app, as well as our recent track record for a similar modification of the apps for EMI delivery, a 6-month time frame is realistic.

Table 1: Functions of Current Apps and Proposed GeoTRAC App

RENEW/MVMNT Apps	Proposed GeoTRAC App
Tracks GPS	Tracks GPS
Creates geofence around places to trigger action from the app	Creates geofence around places to trigger action from the app
Sends daily and place-based surveys and messages	Sends daily and place-based surveys and messages
Provides reminders for appointments, missed surveys	Provides reminders for appointments, missed surveys
Allows participants to contact study team	Allows participants to contact study team
	**Incorporates functionality to take breathalyzer readings, submit results, and see results over time (using existing BACTrack API/development kit)**
	**Includes educational content to support TRAC MI sessions (alcohol triggers diary, summary of drinking reduction goals, guided breathing exercise)**

Reviewers also wanted more information on the range of error of GPS location and geofencing technology. We developed the functionality for using geofencing to trigger assessments and messages for our RENEW/MVMNT apps and have tested it extensively for our 2 R01s, including Beta testing and concurrent monitoring of accuracy for our study participants (i.e., we ask participants at each interview if there were times when they went to locations where they did not receive assessments). We have conducted over 1300 place-based triggered surveys and have received only a small number of reported inaccuracies (<15) in location-based triggered assessments.

Reviewers also wanted clarification on how messaging for the GeoTRAC arm would handle locations that are highly-frequented (e.g., homes) to avoid message overload. For these locations (determined by the initial activity space assessment), we will collect information about specific times when triggers/drinking behavior are highest and will only send messages when they are at those high-frequency locations at those specific times (e.g., at home after 5pm on weekends). Also, to reduce message burden, we will cap messages for a specific location at 2/day and no more often than every 2 hours. Reviewers were also concerned about possible fatigue related to completing twice-daily breathalyzer and survey responses. In our previous studies, with similar frequencies of breathalyzers and surveys over 8 weeks with comparable incentives, we saw high rates of completion (70% of breathalyzers in TRAC; daily survey completion rates >75%), suggesting that we have a track record of conducting these assessments with minimum fatigue.

More information on how we will handle variations in technological expertise and literacy.

We have considerable experience working with populations with varied levels of technological literacy and have developed strong procedures to provide training on the use and navigation of both smartphones and the specific technologic components of our study (e.g., apps, breathalyzer). Participants in each condition will be trained to navigate the app including completing assessments, opening messages, completing breathalyzer readings, and implementing data safety and confidentiality procedures. Wallet sized cards with information from the training will be provided. We will also do regular checks at assessments and MI sessions to allow participants to report problems, and we will complete video calls to troubleshoot if needed. Finally, the app will include functionality for participants to contact the study team if they experience technical difficulties.

More clarity on the description of the content of the risk reduction text messages.

Reviewers wanted more clarity on how intervention message content will be developed. One of the innovations of the current study is that intervention messaging delivered by the GeoTRAC app will be tailored to each individual based on responses from their MI sessions and activity space assessments. Participants will identify which type of triggers they experience at their locations during the activity space assessments and then develop strategies for addressing these triggers during their MI sessions. These strategies will be entered in the back-end of the app for each individual, allowing for tailored messaging when they go to those locations. The messages will generally utilize the following formula: *"Looks like you're at [INSERT LOCATION NAME HERE]. Because sometimes you experience [TYPE OF TRIGGER] here, don't forget that you can [INSERT STRATEGY HERE] if you feel an urge to drink."* Messages will continue to be added as individuals progress through TRAC, incorporating different triggers and strategies identified through their work in the MI sessions.

More information on the unique expertise of the MPIs and previous collaborations of the study team.

Drs. Lauckner and Kershaw bring shared but unique expertise to the proposed project that provides a strong team science model. Dr. Lauckner is an expert on mHealth and telehealth, MI interventions, and

alcohol-reduction interventions, including among people with HIV. Dr. Kershaw has expertise in behavioral HIV prevention interventions, as well as in conducting activity space assessments, GPS tracking, and assessing the impact of geographical context. For this project, Dr. Lauckner will provide key expertise related to the delivery and refinement of the TRAC intervention, which she has tested in her ongoing K01 and R21 grants. Dr. Kershaw will oversee the app development and will provide expertise related to geographic predictors of risk behavior and collection of location-based data. Overall, the team has a history of collaborations. Drs. Lauckner and Kershaw have worked together for the past 6 years (Dr. Lauckner serves as a Co-I on both of Dr. Kershaw's current R01s; Dr. Kershaw serves as a secondary mentor on Dr. Lauckner's K01). Additionally, Dr. Kershaw works with Drs. Boyd and Restar as part of the REIDS Fellowship, a fellowship on HIV equity research for underrepresented scholars that Dr. Kershaw serves as the PI and Director. Dr. Lauckner and Dr. Adatorwovor are both affiliated with the Center for Health Equity Transformation at the University of Kentucky.

More clarity on the HIV related outcomes and how assessments will be administered.

We will assess HIV related outcomes, with our primary outcomes being condomless anal insertive and receptive sex acts, number of partners, and PrEP use and adherence. However, given that HIV risk is influenced by partner characteristics, we will also assess sexual behavior for each reported partner during the study period as well as type of partner (e.g., main, casual, transactional, partner HIV status), allowing us to see if behavior differs for specific partner types. We will also create an unprotected anal insertive and receptive sex acts variable that includes sex without a condom or adherent PrEP use (>90% in the past 30 days).

Reviewers also wanted clarity on how assessments will be administered. Our baseline, post-intervention, 6, and 12 month assessments will be administered using Qualtrics over Zoom. Daily and place-based triggered assessments will be completed by the participants on our app. We have had considerable success doing assessments these ways in the past and have developed comprehensive processes and procedures to implement assessments using both Qualtrics and the app.

No mention of implementation science plans for further scale up or dissemination of the intervention.

Given the complexity of a 3-arm trial, we do not have the capacity for an extensive implementation evaluation, but we are collecting multiple process measures on fidelity, app use, and technologic issues, as well as qualitative assessments from participants on challenges encountered during the interventions and suggestions for improvement and scale-up. If successful, this project will lead to a larger implementation science grant to assess implementing the intervention in different settings and contexts.

Inclusion of conceptual and theoretical concepts in the study design and intervention.

The conceptual model for this study was developed based on tenets of the social-ecological model, which considers the effects of environment on risk behavior, and the transtheoretical model, which informs the MI intervention. The model includes environmental characteristics (e.g., personal history of alcohol use at locations) and real-time factors (e.g, alcohol use of others at locations, alcohol cravings), which will be used to inform discussions on alcohol use triggers and location-based message development, as well as individual characteristics (e.g., drinking refusal self-efficacy, readiness to change), which will be targeted through the MI session content. The constructs within the conceptual model will be measured during baseline and follow-up assessments, and exploratory analyses will explore their effects on alcohol use and HIV risk outcomes.

We hope that this response addresses all of the substantive questions raised by the reviewers. If you have any remaining questions, or if we can facilitate the review process in any way, please do not hesitate to contact us. We are dedicated to conducting the most rigorous study possible to better allow us to develop an innovative intervention that can improve the health and well-being of sexual and gender minority individuals.

References

1. Gilbert PA, Pass, L. E., Keuroghlian, A. S., Greenfield, T. K., & Reisner, S. L. . Alcohol research with transgender populations: A systematic review and recommendations to strengthen future studies. . *Drug and alcohol dependence.* 2018;186:138-146.
2. Hughto JM, Quinn, E. K., Dunbar, M. S., Rose, A. J., Shireman, T. I., & Jasuja, G. K. . Prevalence and co-occurrence of alcohol, nicotine, and other substance use disorder diagnoses among US transgender and cisgender adults. *JAMA network open.* 2021;4:e2036512-e2036512.
3. Delgado JR, Segura, E. R., Lake, J. E., Sanchez, J., Lama, J. R., & Clark, J. L. . Event-level analysis of alcohol consumption and condom use in partnership contexts among men who have sex with men and transgender women in Lima, Peru. *Drug and alcohol dependence.* 2017;170:17-24.
4. Glynn TR, & van den Berg, J. J. . A systematic review of interventions to reduce problematic substance use among transgender individuals: a call to action. . *Transgender health.* 2017;2:45-59.
5. Hughto JM, Restar AJ, Wolfe HL, et al. Opioid pain medication misuse, concomitant substance misuse, and the unmet behavioral health treatment needs of transgender and gender diverse adults. *Drug and alcohol dependence.* 2021;222:108674.
6. Restar A, Ogunbajo A, Adia A, et al. Using structural equation modelling to characterise multilevel socioecological predictors and mediators of condom use among transgender women and cisgender men who have sex with men in the Philippines. *BMJ Global Health.* 2020;5(7):e002463.
7. Restar AJ, Jin H, Ogunbajo A, et al. Prevalence and risk factors of nonmedical prescription opioid use among transgender girls and young women. *JAMA network open.* 2020;3(3):e201015-e201015.
8. Restar AJ, Kuhns L, Reisner SL, Ogunbajo A, Garofalo R, Mimiaga MJ. Acceptability of antiretroviral pre-exposure prophylaxis from a cohort of sexually experienced young transgender women in two US cities. *AIDS and Behavior.* 2018;22(11):3649-3657.
